# Failure of Glial Cell-Line Derived Neurotrophic Factor (GDNF) in Clinical Trials Orchestrated By Reduced NR4A2 (NURR1) Transcription Factor in Parkinson’s Disease. A Systematic Review

**DOI:** 10.3389/fnagi.2021.645583

**Published:** 2021-02-24

**Authors:** Piniel Alphayo Kambey, Kouminin Kanwore, Abiola Abdulrahman Ayanlaja, Iqra Nadeem, YinZhen Du, Wokuheleza Buberwa, WenYa Liu, Dianshuai Gao

**Affiliations:** ^1^Xuzhou Key Laboratory of Neurobiology, Department of Neurobiology and Anatomy, Xuzhou Medical University, Xuzhou, China; ^2^Department of Nephrology, Xuzhou Medical University, Xuzhou, China

**Keywords:** Parkinson’s disease, glial cell line-derived neurotrophic factor (GDNF), nuclear receptor related factor one (Nurr1)-also called Nr4a2, dopamine neurons, transcription factor

## Abstract

Parkinson’s disease (PD) is one of the most common neurodegenerative maladies with unforeseen complex pathologies. While this neurodegenerative disorder’s neuropathology is reasonably well known, its etiology remains a mystery, making it challenging to aim therapy. Glial cell-line derived neurotrophic factor (GDNF) remains an auspicious therapeutic molecule for treating PD. Neurotrophic factor derived from glial cell lines is effective in rodents and nonhuman primates, but clinical findings have been equivocal. Laborious exertions have been made over the past few decades to improve and assess GDNF in treating PD (clinical studies). Definitive clinical trials have, however, failed to demonstrate a survival advantage. Consequently, there seemed to be a doubt as to whether GDNF has merit in the potential treatment of PD. The purpose of this cutting edge review is to speculate as to why the clinical trials have failed to meet the primary endpoint. We introduce a hypothesis, “Failure of GDNF in clinical trials succumbed by nuclear receptor-related factor 1 (Nurr1) shortfall.” We demonstrate how Nurr1 binds to GDNF to induce dopaminergic neuron synthesis. Due to its undisputable neuro-protection aptitude, we display Nurr1 (also called Nr4a2) as a promising therapeutic target for PD.

## Introduction

In recent years, substantial work has been carried out in the area of neurotrophic factors (Ferreira et al., 2018; Skaper, 2018; Stoker and Barker, 2020). The discovery of neurotrophins and the family of glial cell line-derived neurotrophic factor has provided significant insight into the growth, plasticity, neuroprotection, and repair (Bar et al., 1998; Patel and Gill, 2007; Yasuhara et al., 2007; Allen et al., 2013). Glial cell-line derived neurotrophic factor (GDNF) is a potent neurotrophic factor located at p12–p13.1 on chromosome five and comprises two (I and II) promoters and five exons (Baecker et al., 1999). Promoter I is located on exon IV upstream. Promoter II is positioned upstream of exon I and contains two enhancers, two silencers, and numerous binding sites for the transcription factors (Baecker et al., 1999; Airavaara et al., 2011). GDNF is involved in the growth, survival, maintenance of mesencephalic dopamine (DA) neurons as well as regeneration of adult dopamine neurons after damage (Lin et al., 1993; Lapchak, 1996; Lapchak et al., 1997b; Ibáñez and Andressoo, 2017). Just as Isaac Newton once wrote, “If I have seen more, it is by standing on the shoulders of giants;” The milestone that we have achieved so far (in GDNF studies) is due to the outstanding work of the fore legends. In-depth studies on neurodegenerative diseases, especially Parkinson’s disease (PD), have been conducted to date. In toxin-induced models of PD, the GDNF is worthwhile, but clinical results have been disappointing. This review article objectively analyses how GDNF began as a hypothesis and extensively examine its trajectory in clinical trials. We also extrapolate the bias of GDNF in neuroprotection and later give our hypothesis as to why this protein failed to meet the primary endpoint in clinical trials. In support of the idea, we stress the need to switch to/or inject nuclear receptor-related factor 1 (Nurr1; agonist or AAV-Nurr1 vector) before starting GDNF therapy.

## GDNF Milestone Started as a Hypothesis

In 1981, a theory entitled “A coalescing hypothesis for the origin of amyotrophic lateral sclerosis, Parkinsonism, and Alzheimer” was proposed by Appel (1981). This meant that both of these conditions were due to the absence of a “hormone” or growth factor that would normally be secreted and retrograde transferred by the target tissue of damaged neurons after being picked up by the presynaptic terminal. He continued by explaining that, Neurotrophic failure in PD can be characterized by striatal cells’ inability to provide the requisite neurotrophic dopamine hormone with substantia nigra cells subsequently damaged. The hippocampus and cortical cells’ failure to supply the relevant cholinergic neurotrophic hormone can cause Alzheimer’s disease defects. The central nervous system tissue culture provides a clear system for assessing these neurotrophic hormones that might allow testing the theories.

## Glial Cell Line-Derived Neurotrophic Factor Trajectory in Clinical Trials

### It All Began in 1993, a Journey Coupled With Hope and Despair

Dr. Leu-Fen Lin and Dr. Frank Collins, both research scientists at a small pharmaceutical firm named Synergen, isolated a protein called neurotrophic factor derived from glial cells in 1991. Two years later, they shared their discovery in this journal (Lin et al., 1993). In the normal functioning of the brain, glial cells are essential. The first observations of the neuroprotective properties of GDNF in animal models of PD started to be documented 1 year after the original Lin, and Collins GDNF study (Hoffer et al., 1994; Beck et al., 1995; Tomac et al., 1995), and the first report of positive effects in nonhuman primate models of PD was published 1 year later, and rigorous studies followed afterward (Bowenkamp et al., 1995; Gash et al., 1995, 1996, 2005; Sauer et al., 1995; Zhang et al., 1997; Rosenblad et al., 2000; Ai et al., 2003). Amgen (a biotechnology company) immediately began setting up clinical trials to determine whether the neuroprotective properties of GDNF would translate into humans, sensing that they were on to a potentially blockbuster cure for Parkinson’s. Sadly, since it does not infiltrate through the blood-brain barrier, a protective membrane protecting the brain, GDNF cannot be given orally (Grondin et al., 2003; Tosi et al., 2020). Amgen researchers hoped that, through inserting a tube into the ventricular system and then injecting GDNF, it would penetrate and circulate the brain and eventually reach the dopamine neurons. Findings of this attempt indicated that this was not an ideal therapeutic process. The ventricular route of administration was deficient because the GDNF was unable to penetrate so far into the brain and had a minimal effect as a result. Worse still, since the cerebrospinal fluid provides connections to the spinal cord and other central nervous system areas, various side effects, including hyponatremia, fatigue, vomiting, and paresthesia (prickly sensation in peripheral nerves), have been recorded in the 38 research participants who received GDNF (Nutt et al., 2003). The side effects caused Amgen to end the study.

Putamen was considered significant in searching out to another brain structure for GDNF infusion (Cothros et al., 2019). Dopamine neurons in the brain reside in a region called substantia nigra, but they project their fibers (or axons) to many other locations, including putamen, and this is where much of their dopamine is supplied. Comprehending that putamen is where many of the dopamine neurons’ fibers can be located, the investigators in Bristol hoped that by pervading GDNF to the vicinity, they would promote dopamine neurons to generate further fibers. They recruited five people with advanced PD and inserted tiny tubes into the putamen, which would allow the GDNF to be injected into that area. In this study, five participants were treated with GDNF for 1 year. The study results showed that GDNF therapy resulted in a 39% increase in OFF-medication motor abilities (according to the Unified Parkinson’s Disease Rating System, UPDRS), a 61% improvement in how participants viewed their capacity to perform everyday activities. A 64% drop in drug-induced dyskinesia (and not detected off-medication), no significant health side effects (none of the problems mentioned in the first study). Notably, the researchers performed brain imaging tests of these subjects and recorded a 28% rise in striatum dopamine storage after 18 months (Gill et al., 2003).

### Two Years Follow-Up Results

As a 2-year follow-up analysis indicated, the same five patients reported a 57% and 63% increase in their OFF-medication motor and everyday life sub-scores (Unified Parkinson’s Disease Rating Scale) behaviors after 2 years of therapy, the impact of GDNF treatment tended to have long-lasting effects in these persons (Patel et al., 2005). And a case study of one trial patient was also released by the researchers, indicating that the beneficial effects of GDNF were already affecting 3 years after the treatment had stopped being administered (Patel et al., 2013). A post-mortem examination of one participant’s brain showed the regeneration of putamen dopamine fibers (Love et al., 2005). In 2007, “Unilateral intraputamenal glial cell line-derived neurotrophic factor in patients with PD: reaction to 1 year of care and 1 year of withdrawal” was needed for independent replication of the research (Slevin et al., 2007). Ten individuals with Parkinson’s were unilaterally implanted with a tube to administer GDNF to the putamen in this phase I research. This means that GDNF was being treated on only one side of the brain. However, after 12 months of therapy, the OFF- and ON-medication states’ participants increased by 42% and 38% compared to the overall UPDRS rating. The patients were excluded from the GDNF (the sponsor intentionally halted therapy) after 12 months of treatment but were evaluated for a further 12 months. By 9–12 months after quitting GDNF injection, GDNF therapy’s effects were entirely lost (UPDRS ratings had returned to baseline levels). More notably, seven out of 10 participants reportedly developed GDNF antibodies (Slevin et al., 2007).

### Was the Open-Label vs. Double-Blind Considered?

The major concern with the GDNF phase 1 clinical trial was that it was an open-label trial. Patients in the study and the doctors carrying out the study all knew who was receiving the drug. The research was not blind, which opened the door to a significant placebo effect possibility. A double-blind clinical trial for GDNF with 34 participants was launched by Amgen in conjunction with the phase I study. Both the researchers and the volunteers were double-blind and did not know who had GDNF or a control treatment. The technique used a very different pump (compared to the one used in the Bristol study) to inject the GDNF into the brain, and some speculated that this might have led to the result of this study. In June 2004, Amgen declared that its clinical trial investigating the effectiveness of GDNF in the treatment of advanced PD had demonstrated a biological benefit but had not shown any clinical change (compared to placebo treatment) after 6 months of use. This is the press release^1^. The research was stopped by Amgen later that year (in September). Two explanations, they cited: (1) pre-clinical results from nonhuman primates who were treated for 6 months in the highest dose category (followed by a 3-month washout period) revealed a substantial depletion of neurons in the cerebellum (Chebrolu et al., 2006; Luz et al., 2016). (2) In 18 of the 34 participants in the study (four of whom had acquired neutralizing activity) were identified (Tatarewicz et al., 2007). In 2006, the analysis findings were published (Lang et al., 2006a).

The latest Bristol study findings emerged in 2019. In the form of a randomized placebo-controlled, single-center trial sponsored by the UK National Health Service (and funded by Parkinson’s UK and The Cure Parkinson’s Trust), this new double-blind investigation of directly administered GDNF took place. Notably, the main difference from what had happened before with this trial was the use of a fresh delivery device designed to provide the putamen with excellent coverage. This study’s results were published in February 2019 (Whone et al., 2019b), and it revealed that the study did not reach its prescribed primary endpoint. At the end of this double-blind study, all patients were allowed to enroll in an open-label extension study using the same GDNF dose regimen and intermittent infusion parameters as in the initial double-blind study. This open-label extension trial, which also lasted 40 weeks, began before the double-blind parent investigation results were known. The main objective was to obtain longer-term safety data and to collect further exploratory data on the clinical effects of GDNF over a more extended period of repeated tissue exposure (Whone et al., 2019a). All 41 participants in the parent study were enrolled in the extension study, and all were included in the evaluations. Again no significant differences have occurred.

### Was GDNF Gene Therapy a Solution?

In prior clinical trials, GDNF was injected into the brain in the form of protein. The idea came that why should it not be used in the form of a gene; the endpoint would probably have been promising. As most neurotrophic factors are labile agents that do not successfully pass the blood-brain barrier readily, viral vectors have a possible means of transmitting GDNF to degenerating dopaminergic neurons (Piguet et al., 2017; Axelsen and Woldbye, 2018; Niethammer et al., 2018). Previous experiments using adenoviral and adeno-associated viral vectors (AAV) have shown that transmission of the GDNF gene to the nigrostriatal system before a 6-OHDA lesion protects against dopaminergic neuron death in rats (McBride et al., 2003; Mandel et al., 1997; Bensadoun et al., 2000). When all these GDNF clinical trials were going on, other members of the GDNF neurotrophic factor family were being studied in Parkinson’s models especially Neurturin in the context of gene therapy. The first phase of the clinical trial was reported^2^ (trial registration number NCT00252850 by CERE-120 company) and the findings showed that the procedure was safe and well-accepted (Marks et al., 2008), so the company launched a Phase II clinical trial (NCT00400634), and the results were released in 2010 (Marks et al., 2010). In this study, 58 patients from nine sites in the USA participated in the trial between December 2006 and November 2008. In patients treated with AAV2-neurturin, there was no substantial change in the primary endpoint [difference −0.31 relative to control individuals (SE 2.63), 95% CI −5.58–4.97; *p* = 0.91; Marks et al., 2010; Bartus et al., 2013]. In 13 out of 38 patients infected with AAV2-neurturin and four out of 20 control subjects, severe adverse effects occurred. Three patients developed tumors in the AAV2-neurturin group and two in the sham surgery group. In 2015, a longer-term follow-up series of findings was released, with 51 patients involved in the study. No substantial variation was observed in the primary endpoint classes or in the majority of secondary endpoints. Two participants encountered cerebral hemorrhages with intermittent signs. AAV2-neurturin was not linked to any potentially relevant adverse events. Interpretation: the transmission of AAV2-neurturin bilaterally in PD to the putamen and substantia nigra was not superior to sham surgery. The treatment was well accepted, and there were no AAV2-neurturin-related clinically relevant adverse events (Warren Olanow et al., 2015). As a result of this conundrum, substantial reviews and, of course, that enunciate diverse views (Matcham et al., 2007; Kirkeby and Barker, 2019; Paul and Sullivan, 2019; Manfredsson et al., 2020) and editorials (Lang et al., 2006b; Penn et al., 2006) have been unveiled.

## Gdnf is Biased in Neuroprotection

### Lentiviral Nigral GDNF Transmission Does Not Inhibit Neurodegeneration in a Parkinson’s Disease Familial Rat Model

The evolution of genetic animal models of PD has been made possible by the discovery of mutations in the *α*-synuclein gene in rare autosomal dominant variants of hereditary PD (Brundin et al., 2017; Koprich et al., 2017). In rats expressing *α*-synuclein, a selective and progressive loss of nigral dopaminergic neurons associated with dopaminergic striatum denervation has been reported (Ip et al., 2017). The appearance of abundant *α*-synuclein-positive inclusions and extensive neuritic pathology is correlated with neuronal degeneration, thus causing a progressive and selective loss of dopamine neurons (Giasson et al., 2002). This recapitulates the major characteristics of PD. Lo Bianco et al. (2004) injected Lenti-GDNF in substantia nigra 2 weeks before nigral administration of lenti-A30P. Although robust expression of GDNF was observed in the entire nigrostriatal pathway due to retrograde or anterograde transport, in the lentiviral-based genetic rat model of PD, lenti-GDNF did not prevent dopaminergic neurodegeneration induced by *α-synuclein*. These findings suggest that GDNF treatment cannot modulate the cellular toxicity associated with abnormal folded protein accumulation (*α*-synuclein; Lo Bianco et al., 2004; Decressac et al., 2011).

### Nurr1 Mediated Down-Regulation Interferes With GDNF Signaling in Nigral Dopamine Neurons

Nurr1 is strongly expressed in the developing and adult ventral midbrain and is necessary for the acquisition and preservation of the dopaminergic phenotype in nigrostriatal neurons (Zetterstrom et al., 1997; Le et al., 1999b; Jankovic et al., 2005). In the absence of Nurr1, the dopaminergic neuronal markers, tyrosine hydroxylase (TH) and dopamine transporter (DAT), as well as the receptor tyrosine kinase signaling fail to demonstrate the formation of ventral midbrain neurons (Eells et al., 2001; Hermanson et al., 2003; Luo, 2012; Hegarty et al., 2013). In addition to the expression of the DA synthesis and release machinery components, down-regulation of Nurr1, caused by α-synuclein, affected the nigral DA neurons’ ability to respond to GDNF *via* Ret expression regulation (Decressac et al., 2012a). Over-expression of Nurr1 in the infected cells essentially reverses the blockade of the GDNF response, and increased expression of Nurr1 can provide proximity defense of nigral DA neurons against α-synuclein toxicity, even in the absence of exogenously administered GDNF (Decressac et al., 2012a).

### Nigrostriatal GDNF Overexpression Induces a Robust Weight Loss in Both Animal Models and Clinical Trials

While GDNF has gained attention as a protein that may cure PD, a progressive neurological condition, owing to its impact on nigrostriatal DA neurons, there is a biologically significant side effect of GDNF that needs to be reported. Bodyweight loss was a regularly reported side effect along these lines while administering exogenous GDNF intracerebroventricularly to rodents, rhesus macaques, or humans (Lapchak et al., 1997a; Nutt et al., 2003; Su et al., 2009; Whone et al., 2019a,b). The leading cause of weight loss is elusive for GDNF-treated patients. Some studies indicate that GDNF administered intranigrally may gain access to ventricular spaces and distribute to hypothalamic nuclei, where neurotransmission changes influence food intake and may eventually contribute to weight loss (Hudson et al., 1995). A similar effect reported by Lapchak, a study which showed that GDNF spreads through the third ventricle and the hypothalamus from the lateral ventricle to the fourth, and this would suggest that GDNF alters the hypothalamic neurotransmission needed for feeding behavior (Lapchak and Hefti, 1992; Lapchak and Araujo, 1994). The weight loss phenomenon in GDNF treated animals has also been explored in obesity cases (Boston, 2004; Tümer et al., 2006; Manfredsson et al., 2009; Mwangi et al., 2014). Recently, Tümer et al. (2006) demonstrated that recombinant adeno-associated virus (rAAV)-mediated hypothalamic overexpression of GDNF induced substantial weight loss in elderly rats and decreased the trajectory of expected weight gain in young rats, indicating that circuits outside the basal ganglia could be involved in the capacity of GDNF to cause weight loss (Tümer et al., 2006). To foster weight loss in mice and nonhuman primates, GDF15 binds to the GDNF family receptor alpha-like (GFRAL), a distant relative of receptors for a particular class of TGF-β superfamily ligands (Mullican et al., 2017; Saarma and Goldman, 2017). Therefore, the weight loss in PD treated with GDNF may have been due to alteration of the hypothalamic circuitry, resulting in a reduction in food consumption, thus increasing energy expenditure.

## Why has GDNF Failed in Clinical Trials?

### Inadequate Transcription Factor NR4A2 (Nurr1) That Should Bind to GDNF (A New Hypothesis)?

While GDNF has achieved significant results in rodents (Beck et al., 1995; Kozlowski et al., 2000; Rosenblad et al., 2000) and nonhuman primates (Gash et al., 1995, 1996, 2005; Zhang et al., 1997; Ai et al., 2003), it has not given therapeutic benefits in PD patients in clinical trials. Why has it failed? To answer this question, we narrate various hypotheses, henceforth, offer our thoughts. One hypothesis postulates that the GDNF pathway is disrupted due to alpha-synuclein pathology in SNpc neurons of PD patients (Decressac et al., 2011) and that PD patients do not respond to GDNF therapy for this cause. Although this hypothesis is backed by laboratory evidence (Decressac et al., 2012a,b), further experiments are warranted, in particular by studying the brains of PD patients, to show impairments in the GDNF pathway in α-synuclein related cases. Another hypothesis indicates that drug delivery procedures were suboptimal, resulting in restricted dissemination in the brain parenchyma of neurotrophic factors that prevented the likelihood of a therapeutic advantage being observed (Bartus et al., 2015). This hypothesis appears to be confirmed by the study of catheters and infusion protocols used in the GDNF trials, laboratory studies evaluating GDNF delivery protocols in primates (Patel et al., 2005; Salvatore et al., 2006; Bartus et al., 2011), and brains of patients involved in the Neurturin clinical trial (Bartus et al., 2015), but still inconclusive findings obtained in subsequent studies, even after a high dose of GDNF and modification in infusion catheters. Another hypothesis is that nigrostriatal degeneration may have been too advanced for the patients to respond to growth factor therapy in patients selected for clinical trials. Two sets of evidence support this hypothesis. The first is from the Neurturin experiment, in which patients with <5 years of the disease reported better motor recovery (Marks et al., 2010). The second comes from a report by Kordower et al. (2013). Brain study in PD patients found that SNpc cells and putamen dopaminergic terminals appear to be lost during the first 4–7 years of diagnosis. This indicates that the rescue of the nigrostriatal system mediated by GDNF relies on the degree of degeneration (Quintino et al., 2019). To rescue the nigrostriatal system and motor deficits, GDNF had to be present in the striatum such that a significant amount of damaged SNpc neurons remain to respond to therapy.

Another hypothesis is that GDNF failure in clinical trials may have been triggered by an inadequate transcription factor NR4A2 (Nurr1) to bind to GDNF to evoke and defend dopamine neurons. Below, we critically review how the Nurr1 (Nr4a2) transcription factor is essential for GDNF to promote dopaminergic neuron protection.

## NURR 1 at a Glance

NURR1 belongs to the family of transcription factors activated by ligands called nuclear receptors (Zetterstrom et al., 1997; Chu et al., 2002). NURR1 lacks a hydrophobic pocket for ligand binding, unlike most other nuclear receptors, and may therefore act as a nuclear receptor-independent of the ligand (Wang et al., 2003). NURR1 was first seen to be correlated with DA neuron activity by its essential role in the formation of midbrain DA neurons (Jankovic et al., 2005). Nurr1 is expressed early in post-mitotic cells in the ventral midbrain (from embryonic day 10.5 in mice) when they begin to express DA neuron features (Riddle and Pollock, 2003). Heterozygous Nurr1-deficient (Nurr1+/−) mouse DA neurons tend to be more vulnerable to toxic stress, including conditions believed to affect the survival of the DA neuron, such as susceptibility to toxin 1-methyl-4-phenyl-1,2,3,6-tetrahydropyridine (MPTP) and 6-hydroxydopamine (6-OHDA; Eells et al., 2002; Tan et al., 2003; Pan et al., 2008). Besides, in aged Nurr1+/– mice, progressive nigrostriatal dysfunction, a PD feature, has been observed (Jiang et al., 2005). By regulating transcription of the dopaminergic genes TH, DT, vesicular monoamine transporter (VMAT), and RET receptor tyrosine kinase, Nurr1 overexpression guides *in vitro* differentiation of mesodiencephalic dopaminergic neurons (mdDAs; Skerrett et al., 2014).

### Nurr1 Binds to GDNF to Stimulate the Synthesis of Dopaminergic Neuron Genes

**Figure 1:**
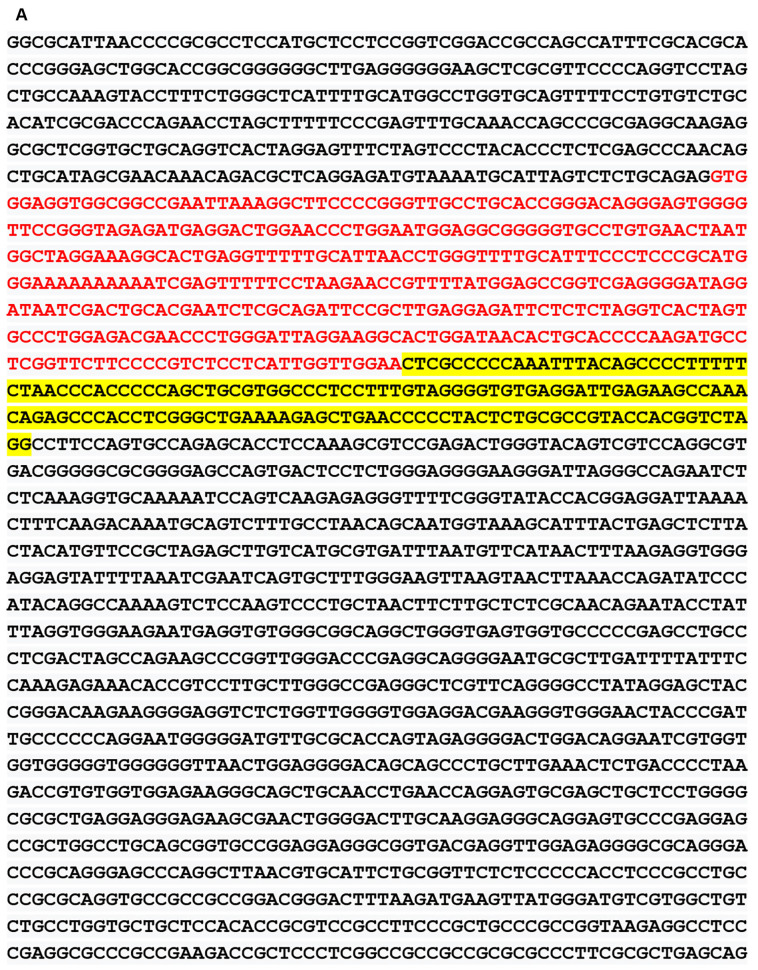

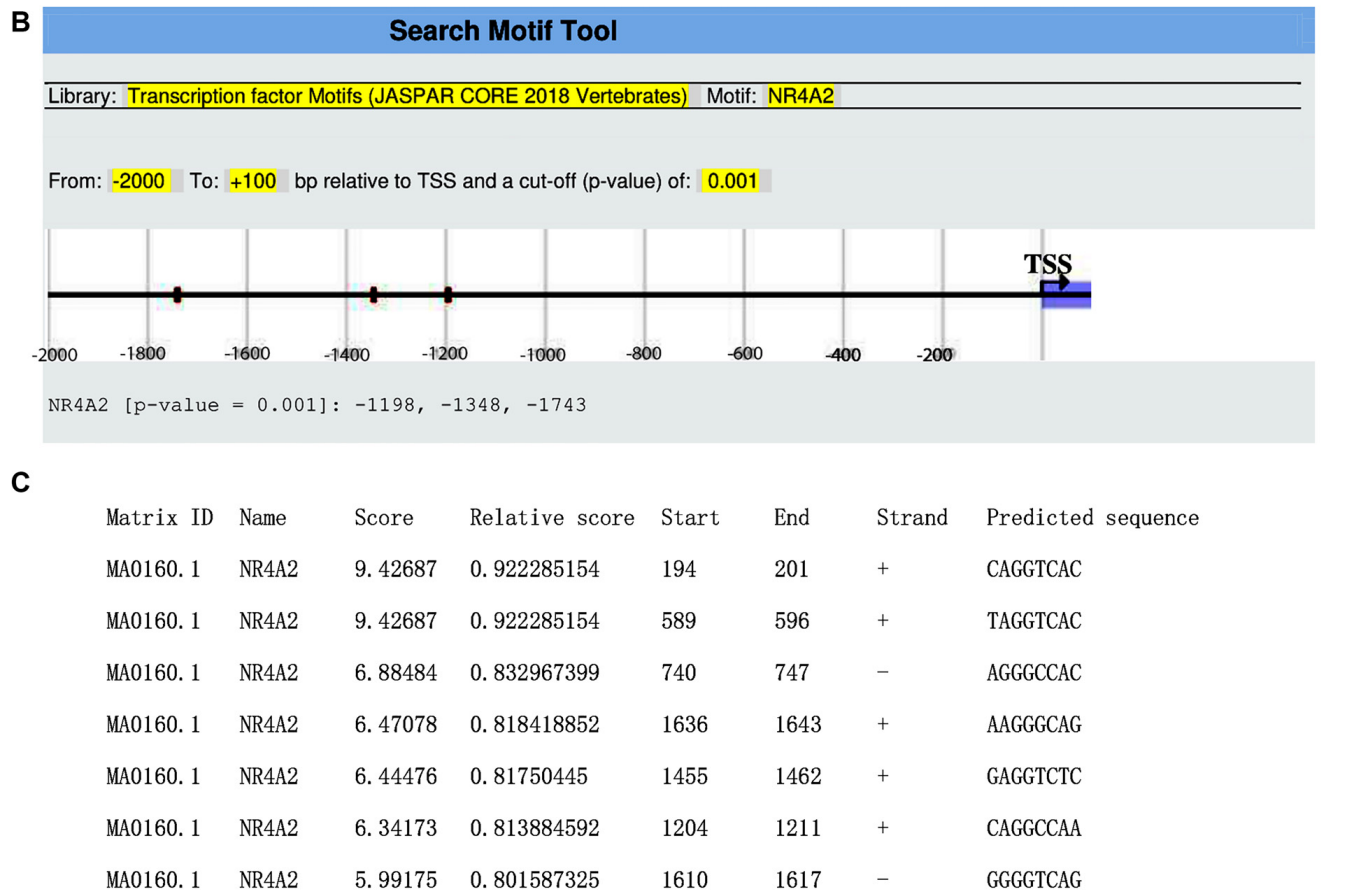
Glial cell-line derived neurotrophic factor (GDNF) promoter from −2,000 bp to +100 bp. **(A)** Red and yellow colors indicate regions with high nuclearreceptor-related factor 1 (Nurr1; NR4A2) transcription factor binding affinity. **(B)** Binding regions, base pairs relative to the transcription start site (TSS) at a cut-off (*p*-value) of 0.001 is indicated. **(C)** The predicted sequence and relative score of NR4A2 binding to GDNF retrieved from http://jaspar.genereg.net/.

**Figure 2:**
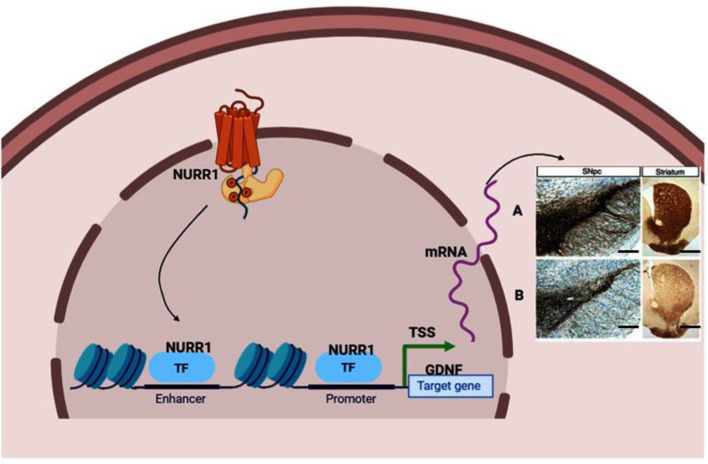
Neuroprotective effect stimulated by transcription factor Nurr1 binding to GDNF promoter. **(A)** Nurr1 binds to GDNF promoter to stimulate the synthesis of dopaminergic neurons marked by tyrosine hydroxylase (TH), dopamine transporter (DAT), and vesicular monoamine transporter (VMAT) neurons in the substantia nigra and fibers in the striatum. **(B)** Inadequate Nurr1 leads to reduced dopamine (DA) neurons and fibers in the substantia nigra and striatum, respectively. Panels **(A,B)** indicate Immunohistochemistry stain (IHC) of TH positive neurons (In the substantia nigra compacta) and fibers (In the striatum). Magnification: 100×, Scale bar: 100 μm. TSS means Transcription start site; TF, Transcription factor. This figure was created with web-based software (www.biorender.com).

We obtained the genomic structure of the human gene GDNF from Gene Bank’s (Ensemble: ENSG00000168621, RefSeq: NM 199231). This promoter is strongly conserved between rats, mice, and humans, and it was earlier identified by Lamberti and Vicini (2014). We were able to classify various GDNF-binding transcription factors using the sequence retrieval tool database^3^. As seen in Figures 1A,B, NR4A42 (Nurr1) was tracked from −2,000 bp to +100 bp and found several binding sites. Then http://jaspar.genereg.net/ on GDNF binding, we obtained the NR4A2 (Nurr1) predicted sequence and relative score (Figure 1C). This demonstrates that there is a close interaction or crosstalk between GDNF and Nurr1 (NR4A2); thus, GDNF activity may be hampered when NR4A2 is physiologically affected. Figure 2 illustrates how NR4A2 (Nurr1) binds to the GDNF promoter to evoke dopaminergic neurons synthesis.

### Nurr1 Regulates Tyrosine Receptor (RET) Expression in Dopamine Neurons of Adult Rat Midbrain (A Key Receptor for GDNF)

Tyrosine receptor is a member of the superfamily of receptor tyrosine kinase and the receptor complex signaling component for glial cell line-derived neurotrophic factor family ligands (Golden et al., 1998; Trupp et al., 1999; Takahashi, 2001). The Ret canonical GDNF receptor, a tyrosine kinase receptor that signals by sarcoma protein (Src)/rat sarcoma (Ras)/mitogen-activated protein kinase (MAPK), phosphatidylinositol-4,5-bisphosphate 3-kinase (PI3K)/Akt, nuclear factor-κ beta (NF-κB), is activated by GDNF (Airaksinen and Saarma, 2002; Paratcha and Ledda, 2008; Kramer and Liss, 2015). In a study done by Drinkut et al. (2016), they observed that even significant overexpression of GDNF in the striatum does not have a neuroprotective or regenerative effect in the absence of Ret in dopaminergic neurons. Therefore, the canonical GDNF receptor Ret appears to be mandatory for mediating the beneficial survival and axonal re-sprouting effect of GDNF (Kowsky et al., 2007; Kramer et al., 2007). Galleguillos et al. (2010) unilaterally knocked-down Nurr1 expression in the substantia nigra (SN) of adult rats using adeno-associated vector to confirm whether Nurr1 controls RET expression during adulthood. A 57.3% drop in Nurr1 mRNA in the SN followed by reduced extracellular DA levels in the striatum was seen in Nurr1 knockdown animals. RET mRNA and protein decreased by 76.9% and 47%, respectively, in the injected SN. This proves that NR4A2 (Nurr1) is paramount for GDNF to mediate its neuroprotective effect *via* the tyrosine receptor.

### Single-Cell Transcriptomics Identifies Nr4a2 (Nurr1) as an Enriched Gene in a Model of Parkinson’s Disease Treated With Stem Cell-Derived Graft

**Figure 3:**
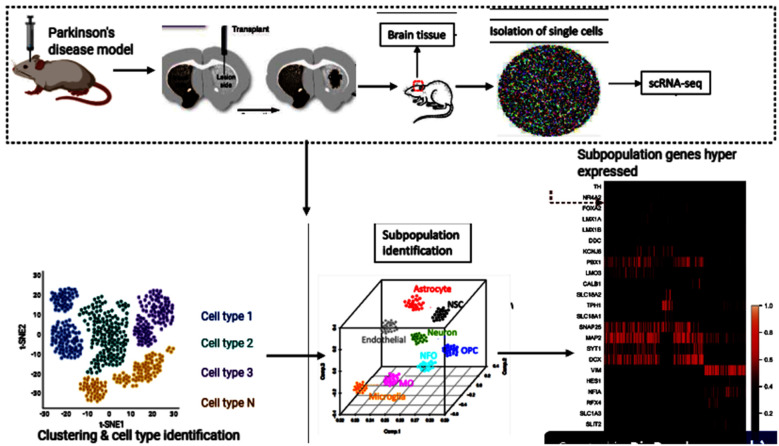
Human embryonic stem cells (hESCs) therapy and single-cell RNA sequencing (scRNA-seq) downstream analyses. This figure was created with web-based software (www.biorender.com).

Single-cell genomics and transcriptomics of single cells have become strong techniques for the genome-wide analysis of single-cell biology. In its transcriptome, epigenome, and local microenvironment, every single-cell in an organism is unique. Because of random fluctuations in the mechanisms driving and regulating transcription and translation, even genetically identical cells exhibit stochastic gene expression (Maleszka et al., 2014; Dey et al., 2015; Pichon et al., 2018). The underlying heterogeneity within cells is a fundamental property of cellular systems for homeostasis and growth (Huang, 2009). Transcriptome spatiotemporal and cell type-specific analyses, the total of all RNA transcripts in a cell or organ, can provide a better understanding of the role of genes in the development and function of the brain and their potential contribution to brain disorders (Keil et al., 2018; Anaparthy et al., 2019). In many brain regions, Single-cell RNA sequencing (scRNA-seq) has allowed researchers to identify various cell subpopulations, pinpoint gene signatures and novel cell markers (Cuevas-Diaz Duran et al., 2017; Ofengeim et al., 2017). Cell replacement has been a long-standing and realistic objective for the treatment of PD (Kim et al., 2020; Parmar et al., 2020). To uncover the previously unknown cellular diversity in a clinically relevant cell replacement PD model, scRNA-seq was used in the experiment by Tiklová et al. (2020b). Human embryonic stem cells (hESCs) were transplanted into the striatum of adult rats that had 6-hydroxydopamine (6-OHDA) unilaterally lesioned. These stem cells gave rise to neuron-rich grafts with innervation extending from the graft core to the dorsolateral striatum and prefrontal cortex. The grafts also contained the expected component of DA neurons as detected 6 months after transplantation by the expression of TH. Furthermore, in animals transplanted with human embryonic stem cell-derived Ventral Midbrain progenitors, paw use and rotational asymmetry induced by 6-OHDA lesions were corrected, confirming functional maturation after transplantation. Gene enrichment had also confirmed that several highly expressed genes were reported, including Nr4a2. Astrocytes, oligodendrocytes, leptomeningeal vascular cells, and neurons were checked for enrichment (Tiklová et al., 2020a; Figure 3). This is the additional evidence that NR4A2 is beneficial and may stand alone as a standard treatment for neurodegenerative disorders, including PD.

### The Lentiviral-Mediated NR4A2 (Nurr1) Genetic Engineering Mesenchymal Stem Cells Protect Dopaminergic Neurons in a Rat Model of Parkinson’s Disease

**Figure 4:**
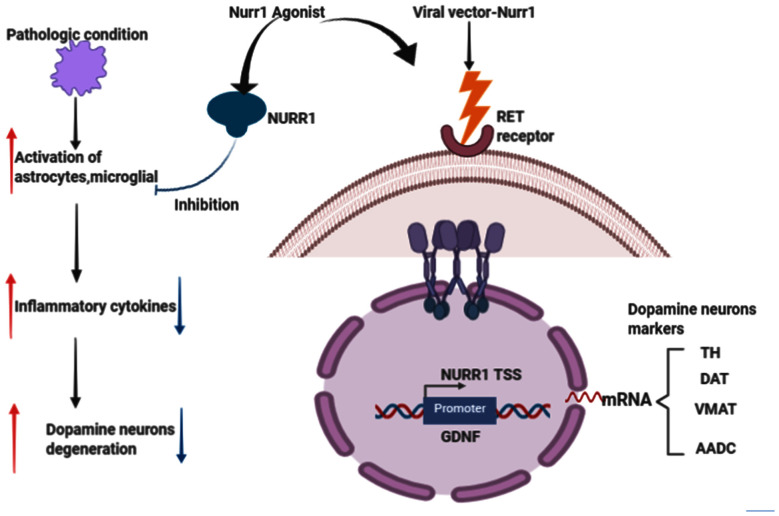
Effects of Nurr1-based therapies in Parkinson’s disease (PD). Nurr1 agonist or Viral vector delivery promotes the synthesis of several genes involved in neurotransmission and dopamine metabolism, including TH, DAT, VMAT, and Aromatic L-amino acid decarboxylase (AADC). Nurr1 treatment also inhibits astrocytes and microglial activation that would eventually cause dopamine neurons degeneration, thus PD. This figure was created with web-based software (www.biorender.com).

Nuclear receptor-related factor 1 (Nurr1) plays a vital role in the growth and maturation of mesencephalic dopamine (DA) neurons (Arenas, 2005; Jankovic et al., 2005; Decressac et al., 2013). It also plays a defensive role in DA neurons’ survival and by inhibiting the activation of microglia and astrocytes (Saijo et al., 2009; Jakaria et al., 2019). Reduced expression of Nurr1 upsurge the susceptibility of MPTP-induced damage of mesencephalic dopamine neurons (Le et al., 1999a). Numerous studies, such as age-based declines in Nurr1 immunoreactivity in human substance nigra (Chu et al., 2002), Nurr1 in PD and related diseases (Chu et al., 2006), decreased NURR1 gene expression in PD3 patients (Le et al., 2008; Liu et al., 2012), decreased NURR1 and Pituitary homeobox 3 (PITX3) gene expression in PD Chinese patients (Liu et al., 2012), decreased Nurr1 mRNA in peripheral blood lymphocytes in PD patients (Montarolo et al., 2016; Li et al., 2018; Yang et al., 2019), are erudite evidence that PD is related to the NR4A2 (Nurr1) shortfall. In the Wang et al.’s (2018) study investigating the therapeutic effects of transplantation of Nurr1 gene-modified mesenchymal bone marrow stem cells (MSCs) into 6-hydroxydopamine (6-OHDA)-induced PD rat models, MSCs was transduced with Nurr1 gene-expressing lentivirus and then transplanted into PD rats intrastriatally. Results revealed that Nurr1 gene-modified MSCs overexpress and *in vitro* secrete Nurr1 protein and also thrive in the brain and migrate. Nurr1 gene-modified MSCs significantly enhanced the pathological activity of PD rats 4 weeks after transplantation and increased the number of TH-positive cells in the substantia nigra (SN) and TH-positive striatum fibers, inhibited glial cell activation and decreased the expression of inflammatory factors in the SN (Wang et al., 2018). Taken together, these results indicate that intrastriatal Nurr1 gene-modified MSCs induced lentiviral vector transplantation has a substantial therapeutic effect for PD rats that could be potentially replicated in humans. Figure 4 describes the Nurr1-based therapies in PD.

### Nurr1 Regulates Dopamine Synthesis and Storage in MN9D Dopamine Cells

The unraveling of dopamine as a neurotransmitter for the brain, its degradation in patients with PD, has been significant revolutionary events in the development of efficient therapy for patients with this condition (Antony et al., 2013). Another compelling solution to symptomatic PD treatment is to entirely rebuild the dopamine production machinery by introducing genes responsible for dopamine synthesis (Drinkut et al., 2012; Aly and Waszczak, 2015; Tenenbaum and Humbert-Claude, 2017). Nurr1 plays an essential role in dopaminergic neuron growth and transcriptional control of AADC, TH, DAT, and VMAT2 (Jankovic et al., 2005). Castro and colleagues showed in their previous research that Nurr1 causes cell cycle arrest and mature morphology (Castro et al., 2001). Hermanson and fellows have shown in another study that Nurr1 increases DA content and aromatic L-amino acid decarboxylase (AADC) and VMAT2 expression in MN9D cells (Hermanson et al., 2003). In addition, AADC and VMAT2 are deregulated in midbrain DA cells of Nurr1 knockout embryos. These together findings provide evidence of an instructive role for Nurr1 in regulating DA synthesis and storage. Therefore a decrease in the level of Nurr1 may impede the impact of GDNF or other GDNF family ligands (GFL) members.

### Nurr1 Pathway for Neuroprotection

**Figure 5:**
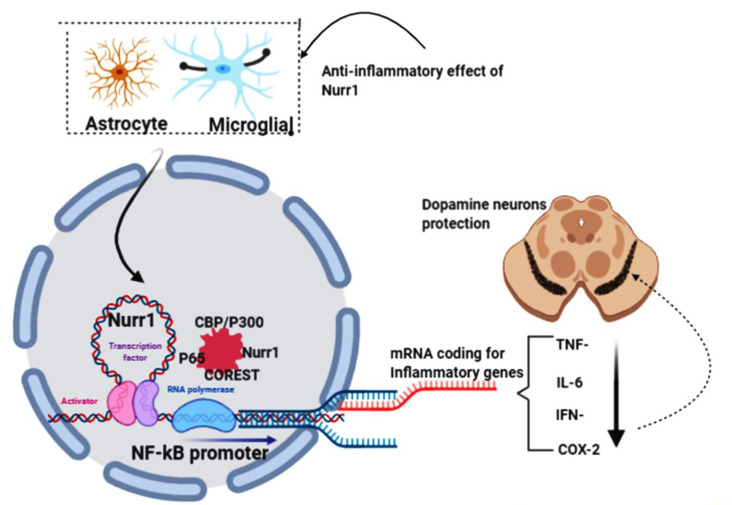
NR4A2 (Nurr1) protects dopamine neurons by impeding neuro-inflammations. In pathological conditions, activated astrocytes and microglial induce the transcription of inflammatory genes known to prompt dopamine neurons’ death in substantia nigra, thus PD. Nurr1, which is highly expressed in astrocytes and microglial, protects dopaminergic neurons by suppressing the transcription of inflammatory genes by binding to the NF-kB promoter in conjunction with coactivator complexes such as p300/CBP. This figure was created with web-based software (www.biorender.com).

Saijo et al. (2009) provided evidence for an unanticipated pathway by which Nurr1 mediates neuroprotection in their eloquently published article. These authors demonstrate that in microglia and astrocytes, mouse Nurr1 works to inhibit the development of inflammatory mediators that cause dopaminergic neurons to die (Saijo et al., 2009). NR4A2 (Nurr1) does not have ligand-binding cavities; instead, it is an immediate-early gene whose expression is caused by several causes, including cyclic AMP, growth factors, hormones, and inflammatory signals. Saijo et al. (2009) discovered a previously unrecognized Nurr1 feature, the suppression of inflammatory gene expression. This suppression defends against the adverse effects of neuroinflammation and indicates new possible pathways that link the role of Nurr1 with PD. By delivering lipopolysaccharide (LPS) to the substantia nigra of mice using stereotaxic injections (a method that uses the coordinate system to target various regions of the brain accurately), the investigators laid the impetus for their discovery. This therapy causes local inflammation and leads to the destruction of tyrosine hydroxylase (TH+) expressing neurons. They proved that Nurr1 expression is caused by inflammation and that local Nurr1 knockdown (by injection of lentiviral vectors expressing short-hairpin RNAs) increases TH+ neuron death. Interestingly, rather than the neurons themselves, the main targets for the neuroprotective effects of Nurr1 tend to be the surrounding microglia and astrocytic cells (Qian et al., 2020). This is implicated in in-vitro studies of microglia and astrocytes in the release of neurotoxic factors causing neuronal death (Polazzi and Monti, 2010). Saijo and colleagues summarize their findings by reiterating that, by docking to NF-κB-p65 in a signal-dependent way on target inflammatory gene promoters, Nurr1 exerts anti-inflammatory effects. Inflammatory signals facilitate the expression of inflammatory genes through the activation of NF-κB signaling and the recruitment to inflammatory promoters of coactivator complexes such as p300/CBP by the NF-κB subunit p65 in conjunction with Co repressor element 1 silencing transcription factor (CoREST) complex (Figure 5).

## Concluding Remarks

GDNF is a potential therapeutic molecule for the treatment of PD. Clinical trials testing GDNF have failed despite an excellent profile in laboratory settings. It is essential to examine why this molecule’s translation from the bench to bedside could not reach the primary endpoint. In the development and survival of dopamine-producing nerve cells in the brain, the nuclear receptor-related protein 1 or Nurr1 (also known as Nr4a2) plays an important role. This protein can regulate dopaminergic neurons’ synthesis by binding to neurotrophic factors such as glial cell line-derived neurotrophic factor. Previous studies have shown that in Parkinson’s patients, Nurr1 is involved in the loss of dopaminergic neurons. Pre-clinical data have shown that raising Nurr1 levels can decrease inflammation and increase neuron survival while declining protein contributes to motor symptoms similar to those seen in PD in rodents and nonhuman primates. Recent evidence from *in vitro* and *in vivo* studies has shown that Nurr1-activating compounds and Nurr1 gene therapy can increase DA neurotransmission and protect DA neurons from environmental toxin-induced cell damage or neuroinflammation mediated by microglia. The pharmacological effects of Nurr1-based PD therapies are: (1) to increase the expression of DA-related genes; (2) to protect or restore DA neurons from neurotoxins; and (3) to prevent the activation of microglia and to suppress neuroinflammation. These robust features make Nurr1 an appealing target for PD treatment. Since the GDNF clinical trials have not yet achieved the desired outcome and based on our speculated hypothesis, “Failure of GDNF in clinical trials succumbed by Nr4a2 (Nurr1) shortfall,” we suggest that the clinical response to GDNF therapy may improve if the nigral or putamen injection is replaced or combined with either the pharmacological Nurr1 agonists or the AAV–NURR1 vector injection.

## Data Availability Statement

The original contributions presented in the study are included in the article, further inquiries can be directed to the corresponding author.

## Author Contributions

PK conceived the idea and drafted this work while KK, AA, IN, YD, WB, WL and DG participated in writing and critically revised this article. All authors contributed to the article and approved the submitted version.

## Conflict of Interest

The authors declare that the research was conducted in the absence of any commercial or financial relationships that could be construed as a potential conflict of interest.

## Footnotes

^1^https://www.amgen.com/media/news-releases/2004/06/amgens-phase-2-study-of-gdnf-for-advanced-parkinsons-disease-fails-to-meet-primary-endpoint-six-months-of-treatment-showed-biological-effect-but-no-clinical-improvement/

^2^https://clinicaltrials.gov/

^3^https://epd.epfl.ch/index.php

## Abbreviations

GDNF: Glial cell-line derived neurotrophic factor
Nurr1: Nuclear receptor related factor one
DA: Dopamine
PD: Parkinson’s disease
AAV: Adeno-associated virus
TH: Tyrosine hydroxylase
DAT: Dopamine transporter
VMAT: Vesicular monoamine transporter
TSS: Transcription start site
ScRNA: seq-Single-cell RNA sequencing
MPTP: 1-methyl-4-phenyl-1,2,3,6-tetrahydropyridine
PITX3: Pituitary homeobox 3
GFL: GDNF family ligands
CoREST: Co repressor element 1 silencing transcription factor
AADC: Aromatic L-amino acid decarboxylase.
